# A survey of *Fusarium* species and ADON genotype on Canadian wheat grain

**DOI:** 10.3389/ffunb.2022.1062444

**Published:** 2022-12-02

**Authors:** Janice Bamforth, Tiffany Chin, Tehreem Ashfaq, Niradha Withana Gamage, Kerri Pleskach, Sheryl A. Tittlemier, Maria Antonia Henriquez, Shimosh Kurera, Sung-Jong Lee, Bhaktiben Patel, Tom Gräfenhan, Sean Walkowiak

**Affiliations:** ^1^ Canadian Grain Commission, Grain Research Laboratory, Winnipeg, MB, Canada; ^2^ Agriculture and Agri-Food Canada, Morden Research and Development Centre, Morden, MB, Canada; ^3^ University of Manitoba, Plant Science, Winnipeg, MB, Canada; ^4^ University of Manitoba, Microbiology, Winnipeg, MB, Canada; ^5^ Julius-Maximilian-University, Core Unit Systems Medicine, Würzburg, Bavaria, Germany

**Keywords:** *Fusarium*, wheat, deoxynivalenol (DON), chemotype, qPCR, grains

## Abstract

**Introduction:**

Wheat is a staple food that is important to global food security, but in epidemic years, fungal pathogens can threaten production, quality, and safety of wheat grain. Globally, one of the most important fungal diseases of wheat is Fusarium head blight (FHB). This disease can be caused by several different *Fusarium* species with known differences in aggressiveness and mycotoxin-production potential, with the trichothecene toxin deoxynivalenol (DON) and its derivatives being of particular concern. In North America, the most predominant species causing FHB is *F. graminearum*, which has two distinct sub-populations that are commonly classified into two main chemotypes/genotypes based on their propensity to form trichothecene derivatives, namely 15-acetyldeoxynivalenol (15-ADON) and 3-acetyldeoxynivalenol (3-ADON).

**Materials and methods:**

We used a panel of 13 DNA markers to perform species and ADON genotype identification for 55, 444 wheat kernels from 7, 783 samples originating from across Canada from 2014 to 2020.

**Results and discussion:**

Based on single-seed analyses, we demonstrate the relationships between *Fusarium* species and trichothecene chemotype with sample year, sample location, wheat species (hexaploid and durum wheat), severity of *Fusarium* damaged kernels (FDK), and accumulation of DON. Results indicate that various *Fusarium* species are present across wheat growing regions in Canada; however, *F. graminearum* is the most common species and 3-ADON the most common genotype. We observed an increase in the occurrence of the 3-ADON genotype, particularly in the western Prairie regions. Our data provides important information on special-temporal trends in *Fusarium* species and chemotypes that can aid with the implementation of integrated disease management strategies to control the detrimental effects of this devastating disease.

## Introduction

Wheat is one of the most widely cultivated cereal crops in the world and is a major contributor towards global food security (Shiferaw et al., 2013). In Canada, wheat is sown across approximately 25 million acres and produces around 35 million tonnes of grain annually, making Canada one of the world’s largest producers of wheat. Grain production and yield are important to meet the food demands of our growing population; however, pests and microbial pathogens can affect wheat production and contribute to yield loss and reduced marketability (Figueroa et al., 2018). While over 30 pests and pathogens have been reported on wheat, Fusarium head blight (FHB) has been the most detrimental to wheat production and quality in Canada over the past three decades (Gilbert and Tekauz, 2000; Simón et al., 2021).

FHB is caused by fungi belonging to the genus *Fusarium* and the disease initiates during wheat flowering (anthesis) (Trail, 2009). Infection by the fungi typically begins in the wheat head or ear, resulting in brownish discolouration at the base of infected glumes, and as the disease progresses through the rachis to adjacent spikelets, the entire wheat head or ear can become prematurely whitened or bleached (Trail, 2009; Brown et al., 2010). Many kernels from the infected plant are either immature or have reduced size and a wrinkled and chalky appearance due to the fungal mycelium. Often, the kernels are also contaminated with mycotoxins that are produced by the fungi, such as the type B trichothecene deoxynivalenol (DON), which pose food safety risks and are regulated in food products (Chen et al., 2021). Losses due to FHB can be severe, particularly in epidemic years such as 2016, where losses in Canada due to reduced yield and mycotoxin contamination resulting from FHB have been estimated at $1 billion (Dawson, 2016).

While disease symptoms on the wheat plant are relatively easy to identify, the disease itself can be caused by several different species of *Fusarium*, each with its own disease and mycotoxin profile. *Fusarium* species such as *F. graminearum*, *F. avenaceum*, *F. acuminatum*, *F. tricinctum*, *F. crookwellense* (*F. cerealis*), *F. culmorum*, *F. equiseti*, *F. pseudograminearum*, *F. langsethiae*, *F. sporotrichioides*, and *F. poae* have all been reported to cause symptoms usually associated with FHB (Beccari et al., 2019; Valverde-Bogantes et al., 2020). In Canada, the occurrence of *Fusarium* species has not just changed over decades but also depends on weather conditions prevailing during growing season. During the 1980s, the species commonly associated with FHB were *Fusarium avenaceum*, *F. acuminatum* and *F. sporotrichioides*, which were prevalent in Manitoba and Saskatchewan, while *F. graminearum* was generally reported in eastern Canada and southern Manitoba but rarely in Saskatchewan or Alberta (Clear and Patrick, 1990). However, these species are considered to be less aggressive than *F. graminearum*, resulting in reduced severity, or percentage of infected seeds when disease occurs (Beccari et al., 2019; Valverde-Bogantes et al., 2020). Since then, there has been a gradual shift away from these species in western Canada and towards *F. graminearum*, which is currently the predominant cause of FHB in Manitoba, Saskatchewan, and Alberta (Kelly et al., 2015; Valverde-Bogantes et al., 2020). In parallel, disease incidence has also been gradually increasing since the 1980s and 1990s, possibly due to the emergence of more aggressive genotypes of *F. graminearum* that are able to outcompete other species and strains (Walkowiak et al., 2015; Valverde-Bogantes et al., 2020).

Many *Fusarium* species are able to produce trichothecene mycotoxins but the chemical structure can vary based on differences in the biosynthetic genes required for their production (Kimura et al., 2007). The most common trichothecene found in Canada is DON but others can be produced, such as nivalenol (NIV), NX, T2 and H-T2 (Kimura et al., 2007; Varga et al., 2015). However, some species, such as *F. avenaceum*, lack the biosynthetic genes and do not produce trichothecenes (Lysøe et al., 2014). Variation in trichothecene production can also occur within species; for example, *F. graminearum* can produce 15-acetyldeoxynivalenol (15-ADON) or 3-acetyldeoxynivalenol (3-ADON) depending on which allele is present for the biosynthetic gene *Tri8*, which encodes a trichothecene C-3 esterase (Alexander et al., 2011). While in both cases, the trichothecene becomes deacetylated *in-planta* to become DON, 15-ADON and 3-ADON producers are distinct mycotoxin phenotypes that belong to different trichothecene chemotypes (Kelly et al., 2015). Given the positive correlation between *F. graminearum* genetic population (i.e. NA1/NA2) and trichothecene chemotype, they could be considered as genotypes as well, with 3-ADON producers (predominantly members of NA2) considered more aggressive than 15-ADON producers (predominantly members of NA1) (Ward et al., 2008; Walkowiak et al., 2015; Oghenekaro et al., 2021).

Disease monitoring programs are critical to understanding the disease and toxin potential of FHB pathogens, and to equip agronomists and plant breeders with the information needed to mitigate the disease. To expand on our monitoring efforts, we have examined individual *Fusarium* damaged kernels (FDKs) to identify the species and trichothecene genotype of *Fusarium*. Traditional identification processes involve growing an axenic isolate of the fungus and the microscopic examination of the pure culture for pigments as well as mycelial and conidial morphology (Summerell and Leslie, 2006). However, with the advent of polymerase chain reaction (PCR) and the availability of whole genome sequences, rapid DNA-based PCR methods have been developed and used for *Fusarium* species and trichothecene genotype determination (Nicholson et al., 1998; Turner et al., 1998; Waalwijk et al., 2004; Wilson et al., 2004; Yli-Mattila et al., 2004; Klemsdal and Elen, 2006; Nicolaisen et al., 2009; Nielsen et al., 2012; Khudhair et al., 2014). Using these assays in nano-well format, we performed DNA-based testing on 55, 444 wheat kernels from 7, 783 samples originating from across Canada from 2014 to 2020. Our survey provides robust data on the occurrence and frequency of the pathogen species and genotypes causing FHB across Canadian provinces and western Canadian crop districts.

## Materials and methods

### Sample collection and quality evaluation

Wheat samples were received as part of the Canadian Grain Commission’s annual Harvest Sample Program (CGC-HSP). Instructions were provided to producers to take many small samples of moving grain during storage bin filling or truck unloading to prepare a bulk sample of approximately 800 g. Bulk samples were cleaned on a Carter Dockage Tester before visually inspected for the presence of FDKs by grain inspectors. The severity of *Fusarium* damage, measured as the mass ratio of FDK in a sample (m/m), was determined for each sample using a subsample of approximately 100 g. The subsample was obtained by dividing the bulk sample using a Boerner divider, to avoid a biased subsample. For samples with trace amounts of FDK, where FHB is present but severity was less than 0.01%, severity was recorded as zero, but FDK were still sampled. All individual FDK were then isolated from the sample for DNA testing, in most cases, multiple FDK were isolated and tested from each sample. In total, 55, 444 wheat kernels from 7, 783 samples were isolated for testing.

### Deoxynivalenol testing

For samples from 2018-2020, samples were cleaned on a Carter Dockage Tester as per the Official Grain Grading Guide of the Canadian Grain Commission (Canadian Grain Commission, 2021a). A Boerner divider was used to prepare a sub-sample of 250 ± 50 g, which was then ground with a Perten Lab Mill 3100. Samples were incrementally sampled to obtain a test portion of 50 ± 0.1 g. Test portions were analyzed for DON using the Neogen Reveal Q lateral flow device and Raptor reader according to the manufacturer’s supplied test instructions. Briefly, ultra-pure water was added to the sample, the mixture was shaken and an aliquot centrifuged, followed by addition with diluent and quantitative analysis. Results below the limit of detection (< 0.3 mg/kg) were recorded as 0 mg/kg, while results above the upper working limit (> 6.0 mg/kg) were recorded as 6.01 mg/kg for statistical analyses.

### DNA extraction of FDK and high-throughput qPCR

FDK isolated from the CGC-HSP were subjected to DNA isolation and high-throughput quantitative PCR (HT-qPCR) for the identification of *Fusarium* species and trichothecene genotypes, which were taken to be analyzed using high-throughput quantitative PCR (HT-qPCR). DNA extraction of FDK from wheat samples was done as described previously (Perry and Lee, 2015). Briefly, kernels were soaked in water overnight, then ground in 96 well plates using a Retsch Mixer Mill (frequency of 30 Hz for 6 minutes). Next, lysis buffer (288 mM NaCl, 10 mM SDS 0.1%, 200 mM Tris-HCl (pH 7.5), 25 mM EDTA) was added to each well, which was followed by additional grinding (frequency of 30 Hz for 1 minute). Samples were then centrifuged and the supernatant were combined with isopropanol for DNA precipitation, which were then resuspended in elution buffer (10 mM Tris-HCl, pH 7.5).

DNA samples were tested for species and trichothecene genotype using HT-qPCR by the Takara SmartChip system. The HT-qPCR system was run using the MyDesign format with a sample count of 216. The primer panel consisted of 13 markers that have been previously reported to be specific to *Fusarium* species or trichothecene genotypes (**Table 1**). For each sample, 6.4 µL of DNA was combined with 6.4 µL of Roche 2X Lightcycler 480 SYBR Green I Master Mix in a microtiter plate, mixed, and centrifuged. Next, 500 nM of primers were combined with the same SYBR Green master mix as above and added to separate wells of the same microtiter plate. After that, as per manufacturers’ instructions (Takara), samples and assays were dispensed into the 5, 184 nanowell reaction chip (SmartChip) using the multi-sample-nano-dispenser (MSND) followed by sealing and loading onto the SmartCycler (Takara) using the protocol: MyDesign_SYBR_GX_Protocol_RevD. Data was exported for further analysis.

**Table 1:** Primer sequences of each *Fusarium* species and ADON genotype used in this study.

Species/genotype	Primer Sequences (5’ to 3’)	Primer reference
*F. avenaceum*	GCTAATTCTTAACTTACTAGGGGCC CTGTAATAGGTTATTTACATGGGCG	Turner et al., 1998
*F. avenaceum or* *F. acuminatum or F. tricinctum*	CCATCGCCGTGGCTTTC CAAGCCCACAGACACGTTGT	Waalwijk et al., 2004
*F. crookwellense (F. cerealis)*	AGTGCCACCATCCCACACG CTGTTCGATGTGAACCAATCGA	Waalwijk et al., 2004
*F. culmorum*	TTGATCAAACCATCATCATC AGAAAGGGTTAGAATCATGC	Klemsdal and Elen, 2006
*F. equiseti*	CACCGTCATTGGTATGTTGTCATC TGTTAGCATGAGAAGGTCATGAGTG	Nicolaisen et al., 2009
*F. graminearum*	ACAGATGACAAGATTCAGGCACA TTCTTTGACATCTGTTCAACCCA	Nicholson et al., 1998
	CCATTCCCTGGGCGCT CCTATTGACAGGTGGTTAGTGACTGG	Nicolaisen et al., 2009
15-ADON	GTTTCGATATTCATTGGAAAGCTAC CAAATAAGTATCGTCTGAAATTGGAAA	Nielsen et al., 2012
3-ADON	AACATGATCGGTGAGGTATCGA CCATGGCGCTGGGAGTT	Nielsen et al., 2012
*F. langsethiae*	CAAAGTTCAGGGCGAAAACT TACAAGAAGACGTGGCGATAT	Wilson et al., 2004
*F. poae*	ACCGAATCTCAACTCCGCTTT GTCTGTCAAGCATGTTAGCACAAGT	Nicolaisen et al., 2009
*F. pseudogramineareum*	CAAGTTTGATCCAGGGTAATCC GCTGTTTCTCTTAGTCTTCCTCA	Khudhair et al., 2014
*F. sporotrichioides*	CCGCGCCCCGTAAAACG ACTGTGTTTGCACACAGATC	Yli-Mattila et al., 2004
*F. tricinctum*	TTGGTATGTTGTCACTGTCTCACACTAT TGACAGAGATGTTAGCATGATGCA	Nicolaisen et al., 2009

### Data analysis

**Figure 1:**
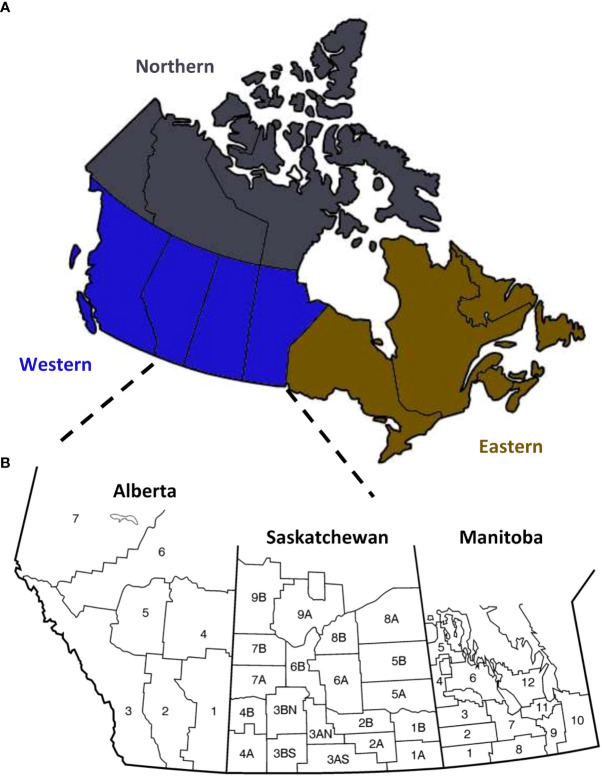
Map of Canada. **(A)** Wheat samples were obtained from western (blue) and eastern (gold) Canada. **(B)** Most of the samples were from the western Canadian provinces of Alberta, Saskatchewan, and Manitoba, which are organized into different crop districts based on growing conditions and geography.

HT-qPCR data was filtered to remove samples with missing data and for data with Ct values greater than 32 (**Table 1**). Wheat samples originating from Ontario, Quebec, and New Brunswick were combined to form one group, eastern Provinces (**Figure 1**). As there is no specific primer targeting *F. acuminatum*, we use the primer for *F. avenaceum* or *F. acuminatum* or *F. tricinctum* as an indirect detection for *F. acuminatum* (**Table 1**; Waalwijk et al., 2004). Since *F. acuminatum* detection is indirect, these identifications are considered putative. If there was a positive amplification for the primer with the *F. avenaceum* primer, it was considered a positive hit for *F. avenaceum*, if there was a positive amplification for the primer with the *F. tricinctum* primer, it is considered a positive hit for *F. tricinctum*. However, if there was a positive amplification only for the non-specific primer capturing *F. avenaceum* or *F. acuminatum* or *F. tricinctum*, it was considered a putative hit for *F. acuminatum*. When examining ADON genotypes of *F. graminearum*, data was subsetted for kernels with *F. graminearum* presence.

Data analysis, organization, and statistics were done using R version 4.1.2 (R Core Team, 2022) and R packages dplyr (Wickham et al., 2014), tidyr (Wickham, 2017), car (Fox et al., 2012), MASS (Ripley, 2011), multcomp (Hothorn et al., 2016), and emmeans (Russell, 2019). Data visualization was done using R packages ggplot2 (Wickham, 2016), ggthemes (Arnold, 2019), ggpubr (Kassambara, 2020), forcats (Wickham, 2019), cowplot (Wilke, 2019), and ggfortify (Horikoshi et al., 2016). As the data distribution was not normal (Shapiro-Wilk test: p< 0.05), non-parametric tests were implemented. A Kendall’s tau correlation test was used to determine the relationship of species presence with FDK severity, which is represented by the percentage of FDK in a sample on a mass basis (**Supplementary Table 1**). Generalized linear models (GLM) were used to assess species presence by year, province, and crop districts. A Poisson distribution with a log link function was used to fit the model, followed by a one-way ANOVA (**Supplementary Table 2**). Species diversity of each sample was determined by Shannon’s diversity index using the R package microbiome (Lahti and Shetty, 2017). Shannon’s diversity index (H) measures species diversity in a sample, by examining the proportion of each species in a sample (*p_i_*):


$$H=\;-\sum p_{i}\times \;\text{ln}(p_{i})$$

For this model, a higher value of the index signifies higher species diversity, while a lower value depicts lower diversity, and zero indicates the presence of one species in the sample. A Wilcoxon rank sum test was used to examine differences between years and provinces. A two-proportion z-test was used to compare proportions of the different ADON genotypes in hexaploid and durum wheat.

### Analysis of environmental data

Historical weather data from Alberta, Manitoba, Saskatchewan, and southwestern crop districts of Saskatchewan were obtained by the publicly available database from Environment Climate Change Canada between 2014-2020 for the months of July and August (Environment and Climate Change Canada, 2022). Mean estimates of total precipitation (P) for each province and for the southwestern crop districts of Saskatchewan were taken across weather stations for July and August of each year.

## Results

Wheat is grown across western and eastern Canada, primarily towards the southern regions (**Figure 1A**); however, most wheat production occurs in the western Canadian provinces of Alberta, Saskatchewan, and Manitoba (**Figure 1B**). We sampled grain across Canada, collected FDK, and tested for the *Fusarium* species and *F. graminearum* trichothecene genotype frequency by HT-qPCR. From 2014 to 2020, between 359 to 1, 808 samples with FDK were assessed each year (**Table 2**). In total, 7, 783 unique samples were tested; 6, 963 were hexaploid wheat, 793 were durum wheat, and 27 were wheat samples where the wheat class was unknown. Most of the samples had multiple FDK, and several kernels from each sample were tested individually for *Fusarium* species and ADON genotype identification, with between 1, 247 to 11, 763 FDK tested each year (**Table 2**). In total, 55, 444 FDK were tested; 47, 923 were hexaploid wheat kernels, 7, 336 were durum wheat kernels, and 185 kernels were from samples where the wheat class was unknown.

**Table 2:** Total number of wheat samples and *Fusarium* damaged kernels (FDK) assessed for each test year, and median FHB severity (% m/m) and mean DON (mg/kg) for wheat samples tested by CGC-HSP beginning from 2018-2020.

Year	Wheat Samples	*Fusarium* damaged kernels (FDK)	FHB Severity (% m/m)	DON (mg/kg)
2014	1, 635	11, 516	1.10 (0-15.9)	–
2015	1, 808	14, 245	0.77 (0-16.2)	–
2016	1, 184	11, 299	1.70 (0.01-100)	–
2018	359	3, 072	0.20 (0.02-2.3)	0.74 (0-5.8)
2019	1, 399	9, 320	0.50 (0-5)	0.43 (0-6)
2020	1, 150	5, 992	0.30 (0-7.5)	0.70 (0-6.01)

In brackets () showing the range of FHB severity and DON for each year.

### *F. graminearum* is the predominant causal agent of FDK in wheat in Canada

**Figure 2:**
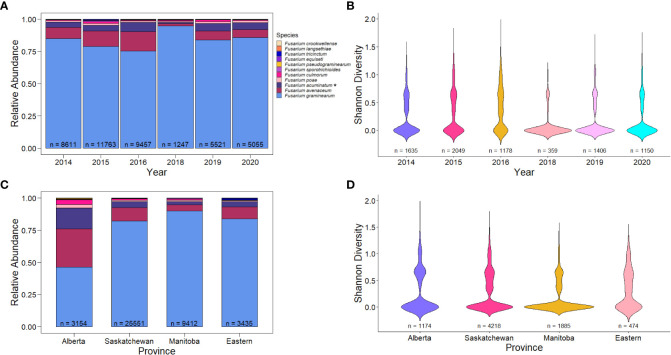
Relative abundance and species diversity of *Fusarium* species in Canada from 2014-2016, 2018-2020. **(A)** Relative abundance of different *Fusarium* species in Fusarium damaged kernels (n) by year. **(B)** Species diversity of different *Fusarium* species in wheat samples with FDK (n) by year. **(C)** Relative abundance of different *Fusarium* species in Fusarium damaged kernels (n) by Canadian provinces. **(D)** Relative abundance of different *Fusarium* species in Fusarium damaged kernels (n) across Canadian provinces. Relative abundance is the proportion of species-specific positive qPCR test results out of the total positive test results. Asterisks (*) signify putative *F. acuminatum*. Species diversity is Shannon’s diversity index based on the proportions of species for each sample.

While there was some variation in *Fusarium* species over the years, the predominant species detected was *F. graminearum* (**Figure 2A**; 81%). Between 75.31% and 94.79% of FDK tested in each year had presence of *F. graminearum*¸ with reduced levels of *F. graminearum* being observed in 2016. Curiously, this is also an epidemic year where high incidence and severity of FHB was reported (**Table 2**) (Dawson, 2016). The second most common species detected was *F. avenaceum* (10.39%), followed by *F. acuminatum* (5.18%). These species were also commonly detected in 2016, where species diversity was highest – i.e. there was the greatest number of unique species present (**Figures 2A, B**; Shannon’s diversity index = 0.813). Some of the most recent years (2018-2020), which had environmental conditions that were less conducive to disease than 2016, had the highest levels of *F. graminearum* and the lowest species diversity within samples (**Figures 2A, B**; p< 0.005, Wilcoxon rank sum test).

**Figure 3:**
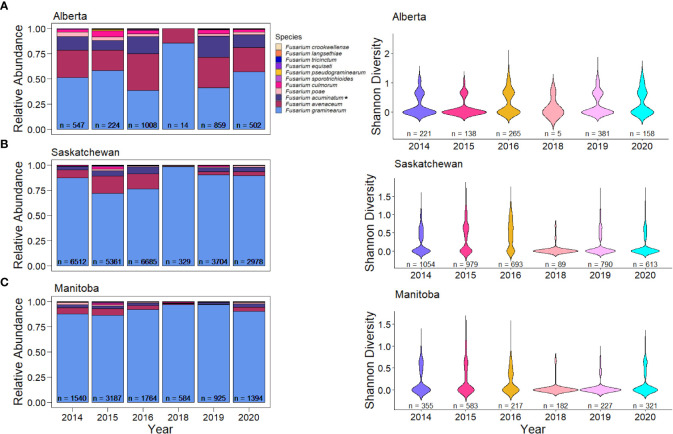
Relative abundance in kernels and species diversity in wheat samples of *Fusarium* species in western Canadian provinces across all years. **(A)** Relative abundance and species diversity of *Fusarium* species in Alberta. **(B)** Relative abundance and species diversity of *Fusarium* species in Saskatchewan. **(C)** Relative abundance and species diversity of *Fusarium* species in Manitoba. Asterisks (*) signify putative *F. acuminatum*.

*Fusarium* species also varied across provinces in Canada. The most pronounced difference in species composition was that of Alberta, which had 46.2% of the samples being *F. graminearum* (**Figure 2C**). In Alberta, we observed increased species diversity (Shannon’s diversity index = 1.308), comprising of 29.9% *F. avenaceum*, 16.23% *F. acuminatum* (putative), 3.58% *F. culmorum*, and 2.76% *F. poae* (**Figure 2C**). This pattern of reduced *F. graminearum* in Alberta was consistent across all years (**Figure 3A**) and crop districts (**Supplemental Figure 1**), and resulted in higher species diversity (**Figure 3B**, **Supplemental Figure 2**). The high sample count and diversity within Alberta in 2016 may be partially responsible for the observed levels of species other than *F. graminearum* in 2016 (**Figure 2A**). Being an epidemic year, where environmental conditions were conducive to infection, disease may have overwhelmed the plants, thereby allowing more opportunity for other species in the region to cause infection. In contrast to Alberta, Manitoba had the highest percentage of *F. graminearum* at 90.02% (**Figure 2C**) and this pattern was consistent across most years (**Figure 3C**) and most crop districts (**Supplemental Figure 1**), resulting in the lowest species diversity (**Figure 3C**, **Supplemental Figure 2**; Shannon’s diversity index = 0.473). Historically, Manitoba also had greater incidence of FHB; this may be in part due to the aggressiveness of *F. graminearum*, which is most common in Manitoba, in conjunction with increased moisture conditions relative to Alberta and southwestern Saskatchewan. When looking more closely within crop districts, we observed that some of the crop districts in southwestern Saskatchewan, such as 4A, 4B, 3BN, and 3BS also had much lower levels of *F. graminearum* that were similar to Alberta, which also shares similar growing environments (**Supplemental Figure 1**; **Supplemental Figure 2**).

While overall levels were low (< 1% of FDK analyzed), we did detect some of the least common causes of FDK, such as *F. langsethiae*, *F. equiseti*, *F. pseudograminearum*, *F. crookwellense*, *F. tricinctum*, and *F. sporotrichioides*, but our results indicate that these are not common on Canadian wheat grain (**Figures 2A, C**). Many of these other species were detected in eastern Canadian provinces, as well as parts of Alberta and Saskatchewan, which likely contributed to the broader diversity observed in those samples (**Figures 2B, D**, **Supplemental Figure 2**).

In summary, it is possible that the diversity observed in each year is rooted in a combination of many factors, including the overall disease incidence and health of the host plants, geographic location and previous inoculum levels, the environmental conditions during the establishment and progression of disease, the genetics and level of resistance of the cultivars being grown, and other on-farm disease management practices (Gilbert and Haber, 2013). While *F. graminearum* remains the dominant species in most growing regions, other species continue to persist in the environment.

### Variation in mycotoxin accumulation and trichothecene genotypes

**Figure 4:**
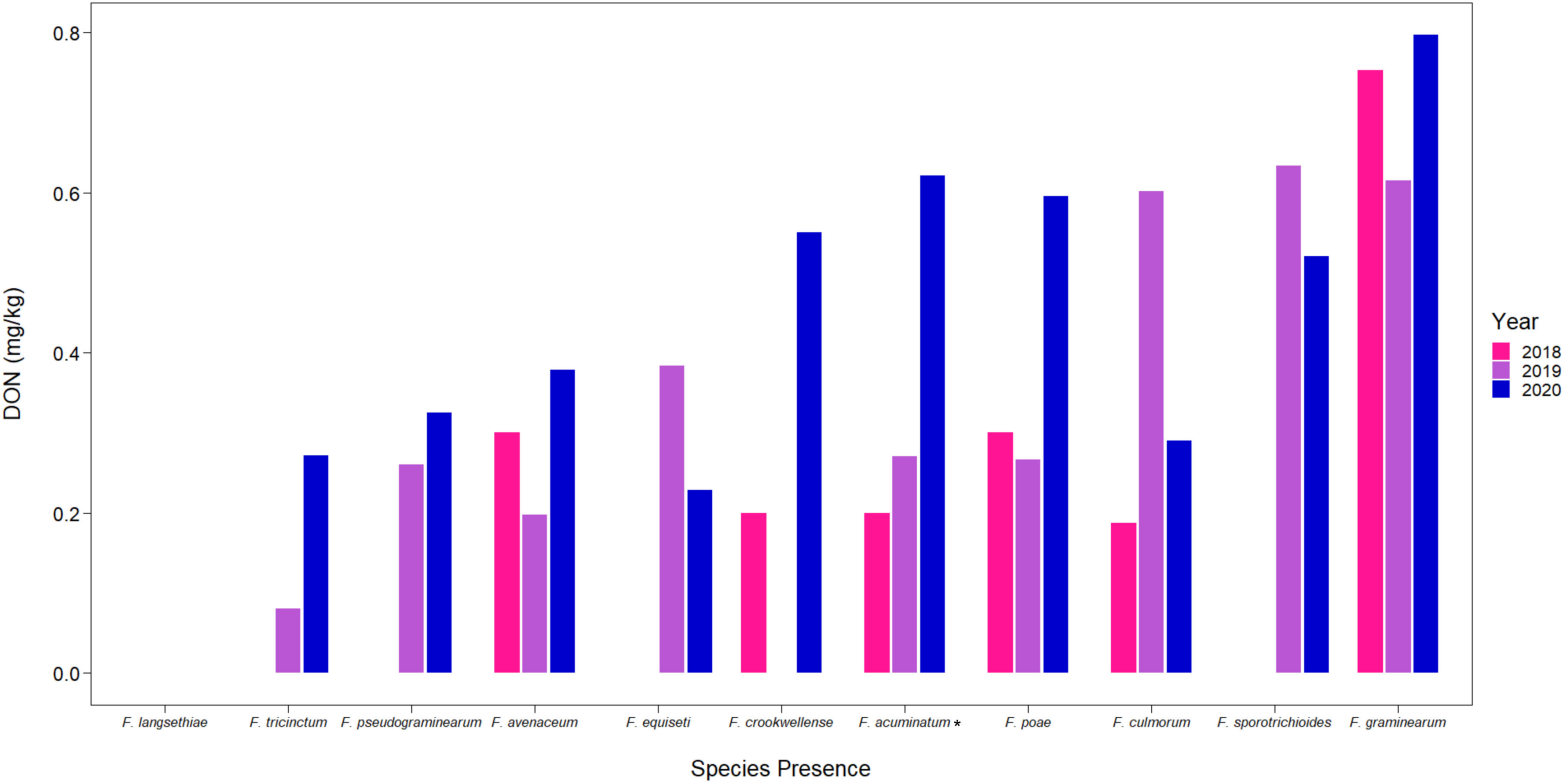
Mean DON (mg/kg) detected from CGC-HSP by different *Fusarium* species present in wheat samples. Asterisks (*) signify putative *F. acuminatum*.

Samples from the CGC-HSP survey from 2018 to 2020 containing kernels that tested positive for the different *Fusarium* species were also analyzed for accumulation of DON. While DON concentrations were generally low, with annual average DON concentrations from the samples tested in this study ranging from 0.43 to 0.74 mg/kg (**Table 2**), we observed differences in DON concentrations based on the *Fusarium* species we detected in the sample (**Figure 4**). On average, samples containing *F. graminearum* had the greatest concentrations of DON, followed by other known trichothecene producers, such as *F. sporotrichioides* and *F. culmorum* (Kimura et al., 2007). Samples containing species that are not known to produce trichothecenes, such as *F. avenaceum*, had much lower concentrations of DON (**Figure 4**) (Lysøe et al., 2014). However, the bulked samples often contained several FDK, including those that were infected by other species that do produce DON; as a result, many of these samples still had detectable levels of DON.

**Figure 5:**
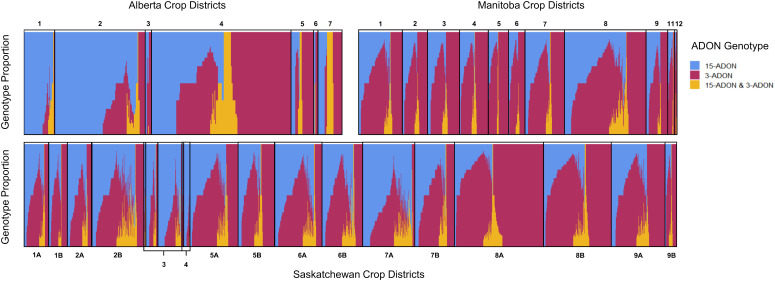
Proportion of ADON genotypes in each sample by western Canadian crop district. Each vertical bar represents a single sample where multiple FDK were tested. Samples corresponding to crop districts in Alberta (top left), Manitoba (top right), and Saskatchewan (bottom) contained kernels that were positive for 15-ADON (blue), 3-ADON (red), or both genotypes (yellow).

**Figure 6:**
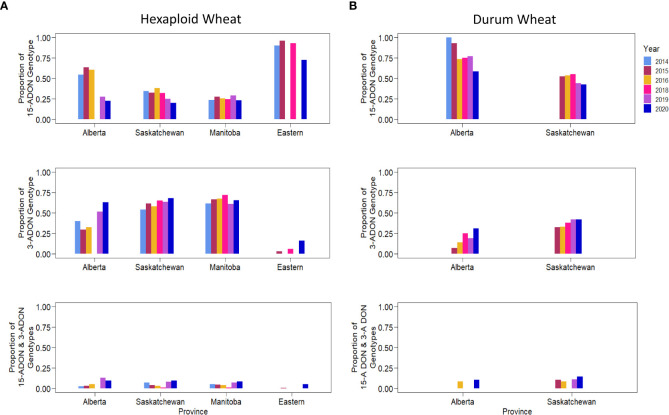
The proportion of different trichothecene genotypes present in wheat kernels across years. The proportion of kernels testing positive for 15-ADON (top), 3-ADON (middle), or both (bottom) for **(A)** hexaploid wheat, and **(B)** durum wheat.

**Figure 7:**
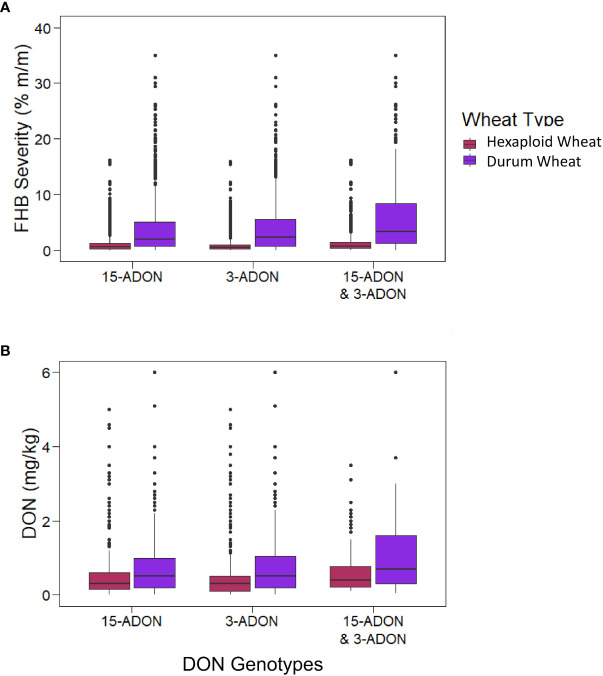
The FHB severity and DON levels for samples with kernels testing positive for different ADON genotypes. The boxes represent the interquartile range (IQR; 25% percentile, median, 75% percentile), the whiskers representing the highest and lowest values within the boundaries of 1.5 × IQR, and dots above the box and whisker plots signify outliers. **(A)** FHB severity (%) for samples containing kernels testing positive for either or both ADON genotypes. **(B)** DON (mg/kg) for samples containing kernels testing positive for either or both DON genotypes.

Kernels containing *F. graminearum* were also tested using HT-qPCR to determine if they belonged to either the 15-ADON or 3-ADON genotype (**Figure 5**). Generally, the proportion of the genotypes in each sample varied by location, with crop districts in Manitoba and Saskatchewan having a predominance of the 3-ADON genotype (**Figure 5**). Isolates that produce 3-ADON have been previously reported to be, on average, more aggressive than those that produce 15-ADON and may be outcompeting other isolates as the population that produces 3-ADON expands westward (Ward et al., 2008; Walkowiak et al., 2015). We observed a trend where the proportion of samples testing positive for 15-ADON declined over our testing period, particularly in Alberta, Saskatchewan, and eastern Canada (**Figure 6**). This was met with a corresponding increase in the number of samples testing positive for the 3-ADON genotype (**Figure 6**). However, we observed that the 15-ADON genotype is the most predominant ADON genotype in eastern Canada (**Figure 6**). Given that the 3-ADON genotype has been present in eastern Canada for over a decade, it is possible that the correlation between genetic population of *F. graminearum* and trichothecene genotype is more balanced and that interactions between the pathogen, a broader range of host crops, and the environment are contributing to population dynamics between chemotypes (Ward et al., 2008; Gilbert et al., 2014). Many samples contained kernels that tested positive for either ADON genotypes (52.55%), suggesting that both chemotypes are often coexisting in the same field; however, only 3.6% of all FDK analyzed kernels tested positive for both ADON genotypes, suggesting that while both are present in the field, one may be dominating on a given plant/kernel (**Figures 5**, **6**). In general, there was no difference in the severity of infection of a sample nor the amount of DON detected in relation to the ADON genotype(s) present on the FDK in samples (**Figure 7**).

### Differences in *Fusarium* species and ADON genotypes between hexaploid and durum wheat

**Figure 8:**
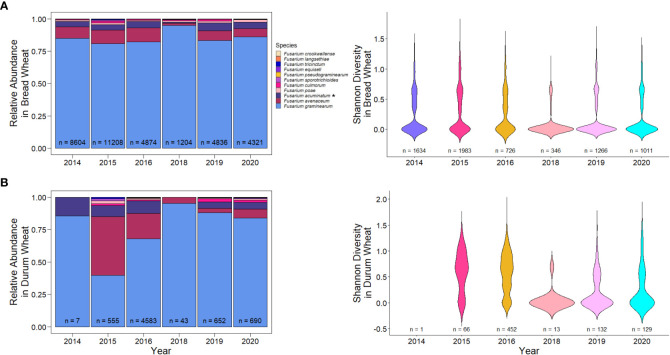
Relative abundance and diversity of *Fusarium* species in **(A)** hexaploid and **(B)** durum wheat across years. Asterisks (*) signify putative *F. acuminatum*.

While hexaploid wheat is grown across western and eastern Canadian provinces, durum wheat is mostly grown in the drier regions of Alberta and southwestern Saskatchewan. As a result, there is a combination of factors that may be contributing to *Fusarium* species and ADON genotype distribution, disease incidence and severity, and DON accumulation. In general, there was higher *F. graminearum* levels in hexaploid wheat compared to durum wheat, particularly in 2015 and 2016, with higher levels of *F. avenaceum* and *F. accuminatum* in durum wheat (**Figure 8**). This may partially explain the increased levels of these species in Alberta and southwestern Saskatchewan, which is the primary growing region of durum (**Figure 2**; **Supplemental Figure 1**). It may also partially explain the observed levels of species other than *F. graminearum* in 2016, the FHB epidemic year in Canada (**Figure 3**). It is unclear why durum wheat has higher levels of species other than *F. graminearum* when compared to hexaploid wheat. While durum wheat is known to be more susceptible than hexaploid wheat to FHB, the different environmental growing conditions and inoculum levels of fungal species in those areas likely also have a role in disease incidence (Haile et al., 2019). Inoculum levels from previous crops, like peas and lentils, in the main growing areas of durum wheat might contribute the high variation of *Fusarium* species and the high levels of *Fusarium avenaceum*. *F. avenaceum* is the main causal agent of root rot diseases in peas and lentil in western Canada (Chatterton et al., 2019).

Previously, it was reported that the *F. graminearum* ADON genotype had an effect on aggressiveness towards durum and hexaploid wheat, where the 3-ADON genotype was more aggressive on hexaploid wheat, and the 15-ADON genotype was more aggressive on durum wheat (Ruan et al., 2021). We observed hexaploid wheat to have a higher proportion of the 3-ADON genotype than durum wheat (p< 0.05, two-sample proportion z-test), and durum wheat to have higher proportion of the 15-ADON genotype than hexaploid wheat (p< 0.05, two-sample proportion z-test). However, our results suggest that there is no difference in the level of FHB severity between samples that contained either or both ADON genotypes with respect to hexaploid wheat or durum wheat (**Figure 7A**). We hypothesize that the growing regions of Alberta and southwestern Saskatchewan, where durum is grown, are still undergoing a shift in pathogen population towards the NA2 population (predominantly 3-ADON producers). This is supported by a temporal shift, where in both hexaploid wheat and durum wheat, we are seeing an increase in the levels of the 3-ADON genotype across sample years (**Figure 6**). It is possible that differences in isolate aggressiveness towards cultivars and species exist, but this may be difficult to parse when the environments and inoculum levels also vary. One difference we did observe was an increase in both FHB severity and DON in durum wheat relative to hexaploid wheat, supporting a known gap in resistance that exists in durum wheat, which we observe seems to be independent of ADON genotype (p< 0.05; two-sample proportion z test) (**Figure 7**). Nevertheless, from a breeding and disease management perspective, cultivar specific differences in infection likely exist and should be continued to be explored.

## Discussion

Pathogen populations and disease pressure are constantly in flux as new species and strains emerge, on-farm management practices change, new cultivars are registered, and climate changes. FHB is an important disease of cereals, affecting grain yield, quality and food safety. Our study provides a robust outlook into the current status of *Fusarium* species and chemotype distribution in Canada for this important disease across years and locations/environments (**Figure 1**; **Supplementary Table S3**, **Supplementary Figure 3**).

In the 1980s, FHB was rarely observed, but when it was, species such as *F. avenaceum*, *F. acuminatum*, and *F. sporotrichioides* were more frequently reported, which eventually shifted towards *F. graminearum* (Clear and Patrick, 1990; Turkington et al., 2011). In Alberta, *F. graminearum* remained uncommon, even into the 2000s (Turkington et al., 2011). We report that the predominant species now found in all Canadian growing regions, including Alberta, is *F. graminearum*. However, other species remain common across Canada and species diversity was greatest in Alberta and in southwestern Saskatchewan. These species are not new to Canada and are available through the culture collections at the Canadian Grain Commission as well as the national collection at Agriculture and Agri-Food Canada (DAOM).

The greater species diversity in Alberta and southwestern Saskatchewan is interesting and is likely due to a combination of several factors. Historically, these areas experience less FHB incidence, especially compared to Manitoba; which may have delayed the shift towards higher incidence of *F. graminearum*. The reduced disease in these areas is likely, in part, due to lower precipitation, since increased moisture and humidity are major contributors to FHB (**Supplementary Table 3**, **Supplementary Figure 3**) (Trail, 2009). Since FHB severity is lower in these areas, it is also preferred for the production of durum wheat, which is generally more susceptible to infection than hexaploid wheat. The most common varieties for hexaploid and durum wheat currently grown in Canada are AAC Brandon (moderately resistant to FHB) and Transcend (moderately susceptible to FHB), respectively (Canadian Grain Commission, 2021b). We also observed increased *Fusarium* species diversity in durum wheat when compared to hexaploid wheat, but this may also be due to growing location and inoculum availability.

In terms of *F. graminearum* ADON genotype, we observed a trend of a continued shift towards the 3-ADON genotype, which is now the most dominant ADON genotype in western Canada, though the 15-ADON genotype is more common in eastern Canada. While occurrence of the 3-ADON genotype is increasing, we did not observe a corresponding increase in FHB severity or DON associated with the 3-ADON genotype. Note that other trichothecenes were not tested in this study as they are seldom associated with *F. graminearum* and are not as abundant in Canadian wheat as DON. Previously, reports have indicated that isolates that produce 3-ADON are more aggressive and produce more trichothecenes *in planta*; however, our results indicate that this may not be the case when averaged across many field environments/locations, years, and cultivars (**Supplementary Figures 3, 4**; **Supplementary Tables 3, 4**) (Walkowiak et al., 2015; Ruan et al., 2021). This suggests that there may be specific host genotype by pathogen genotype by environment interactions that are contributing to situation specific differences in disease and toxin production that warrants further investigation.

Our study was able to uniquely study large numbers of samples using PCR-based methods. The method we used has advantages over traditional microscopy and morphology based methods in terms of cost and throughput. However, in large-scale studies such as ours, the possibility also exists for errors that may arise from primer specificity or DNA quality. While our study provides support for previously reported trends of increasing levels of *F. graminearum* and the 3-ADON chemotype, it is also important to complement such genotypic studies with additional validation studies. This may be particularly important for some species, such as *F. langsethiae*, that were only detected in a few FDK out of the 55, 444 tested, as well as *F. acuminatum*, for which we performed indirect putative identifications.

In summary, it is likely that the species and chemotype diversity we observed in each year and location is rooted in a combination of many factors, including the overall disease incidence and health of the host plants, inoculum levels, the environmental conditions during the establishment and progression of disease, the genetics and level of resistance of the cultivars being grown, and other on-farm disease management practices (Gilbert and Haber, 2013). While reduced moisture and precipitation in some regions may have delayed shifts in species and chemotypes, these shifts appear to be on-going. In years where precipitation is high, there is also the possibility of widespread epidemics, such as in 2016 (**Supplementary Figure 3**; **Supplementary Table 3**). It is therefore prudent to continue to develop new cultivars that are resistant to the latest pathogen populations. Disease monitoring and targeted studies into the factors contributing changes in pathogen populations are important to equip producers, agronomists, and breeders with the latest targets for disease management, particularly in the context of intensified agriculture and climate change.

## Data availability statement

The original contributions presented in the study are included in the article/**Supplementary Material**. Further inquiries can be directed to the corresponding authors.

## Author contributions

JB, TA, and TC performed technical experimentation and formal analyses. KP contributed data and performed manuscript editing. ST, MH, NG, SK, S-JL, and BP provided technical support and performed manuscript editing. TC and SW prepared the original manuscript draft and all visualizations. TG and SW designed the project and performed project supervision. All authors contributed to the article and approved the submitted version.

## Acknowledgments

We would like to acknowledge all the hard work by those involved in the Harvest Sample Program, from the Inspection Services division of the Canadian Grain Commission, the Analytical Services unit within the Grain Research Laboratory, and the producers who participated in the program.

## Conflict of interest

The authors declare that the research was conducted in the absence of any commercial or financial relationships that could be construed as a potential conflict of interest.

## Publisher’s note

All claims expressed in this article are solely those of the authors and do not necessarily represent those of their affiliated organizations, or those of the publisher, the editors and the reviewers. Any product that may be evaluated in this article, or claim that may be made by its manufacturer, is not guaranteed or endorsed by the publisher.

## References

[B1] Alexander N. J. McCormick S. P. Waalwijk C. van der Lee T. Proctor R. H. (2011). The genetic basis for 3-ADON and 15-ADON trichothecene chemotypes in fusarium. Fungal Genet. Biol. 48 (5), 485–495. doi: 10.1016/j.fgb.2011.01.003 21216300

[B2] Arnold J. B. (2019). “Ggthemes: Extra themes, scales and geoms for ‘ggplot2’,” in R package version, vol. 4. .

[B3] Beccari G. Arellano C. Covarelli L. Tini F. Sulyok M. Cowger C. (2019). Effect of wheat infection timing on fusarium head blight causal agents and secondary metabolites in grain. Int. J. Food Microbiol. 290, 214–225. doi: 10.1016/j.ijfoodmicro.2018.10.014 30366263

[B4] Brown N. A. Urban M. van de Meene A. M. L. Hammond-Kosack K. E. (2010). The infection biology of fusarium graminearum: Defining the pathways of spikelet to spikelet colonisation in wheat ears. Fungal Biol. 114 (7), 555–571. doi: 10.1016/j.funbio.2010.04.006 20943167

[B5] Canadian Grain Commission (2021a) Official grain grading guide (Winnipeg MB: Canadian Grain Commission). Available at: https://www.grainscanada.gc.ca/en/grain-quality/official-grain-grading-guide/ (Accessed 2022 Apr 4).

[B6] Canadian Grain Commission (2021b) Grain varieties by acres insured. Available at: https://www.grainscanada.gc.ca/en/grain-research/statistics/varieties-by-acreage/ (Accessed 2022 Jun 3).

[B7] Chatterton S. Harding M. W. Bowness R. McLaren D. L. Banniza S. Gossen B. D. (2019). Importance and causal agents of root rot on field pea and lentil on the Canadian prairies 2014–2017. Can. J. Plant Pathol. 41 (1), 98–114. doi: 10.1080/07060661.2018.1547792

[B8] Chen C. Frank K. Wang T. Wu F. (2021). Global wheat trade and codex alimentarius guidelines for deoxynivalenol: A mycotoxin common in wheat. Global Food Secur. 29, 100538. doi: 10.1016/j.gfs.2021.100538

[B9] Clear R. M. Patrick S. K. (1990). Fusarium species isolated from wheat samples containing tombstone (scab) kernels from ontario, manitoba, and saskatchewan. Can. J. Plant Sci. 70 (4), 1057–1069. doi: 10.4141/cjps90-128

[B10] Dawson A. (2016). Fusarium Conference Hears of Disease Resurgence. Alberta: Manitoba Co-operator. Available at: https://www.manitobacooperator.ca/news-opinion/news/2016-spring-wheat-crops-on-the-prairies-hit-hard-by-fusarium/.

[B11] Environment and Climate Change Canada (2022) Monthly climate summaries (Accessed 2022 Oct 29).

[B12] Figueroa M. Hammond-Kosack K. E. Solomon P. S. (2018). A review of wheat diseases-a field perspective. Mol. Plant Pathol. 19 (6), 1523–1536. doi: 10.1111/mpp.12618 29045052 PMC6638159

[B13] Fox J. Weisberg S. Adler D. Bates D. Baud-Bovy G. Ellison S. . (2012). Package ‘car’ Vol. 16 (Vienna: R Foundation for Statistical Computing).

[B14] Gilbert J. Brûlé-Babel A. Guerrieri A. T. Clear R. M. Patrick S. Slusarenko K. . (2014). Ratio of 3-ADON and 15-ADON isolates of fusarium graminearum recovered from wheat kernels in Manitoba from 2008 to 2012. Can. J. Plant Pathol. 36 (1), 54–63. doi: 10.1080/07060661.2014.887033

[B15] Gilbert J. Haber S. (2013). Overview of some recent research developments in fusarium head blight of wheat. Can. J. Plant Pathol. 35 (2), 149–174. doi: 10.1080/07060661.2013.772921

[B16] Gilbert J. Tekauz A. (2000). Review: Recent developments in research on fusarium head blight of wheat in Canada. Can. J. Plant Pathol. 22 (1), 1–8. doi: 10.1080/07060660009501155

[B17] Haile J. K. N’Diaye A. Walkowiak S. Nilsen K. T. Clarke J. M. Kutcher H. R. . (2019). Fusarium head blight in durum wheat: Recent status, breeding directions, and future research prospects. Phytopathology® 109 (10), 1664–1675. doi: 10.1094/PHYTO-03-19-0095-RVW 31369363

[B18] Horikoshi M. Tang Y. Dickey A. (2016) Ggfortify: Data visualization tools for statistical analysis results. Available at: https://CRAN.R-project.org/package=ggfortify.

[B19] Hothorn T. Bretz F. Westfall P. Heiberger R. M. Schuetzenmeister A. Scheibe S. . (2016). “Package ‘multcomp’. simultaneous inference in general parametric models,” in Project for statistical computing(Vienna, Austria).

[B20] Kassambara A. (2020). Package ‘ggpubr’. r package version 0.1 6.

[B21] Kelly A. C. Clear R. M. O’Donnell K. McCormick S. Turkington T. K. Tekauz A. . (2015). Diversity of fusarium head blight populations and trichothecene toxin types reveals regional differences in pathogen composition and temporal dynamics. Fungal Genet. Biol. 82, 22–31. doi: 10.1016/j.fgb.2015.05.016 26127017

[B22] Khudhair M. Melloy P. Lorenz D. J. Obanor F. Aitken E. Datta S. . (2014). Fusarium crown rot under continuous cropping of susceptible and partially resistant wheat in microcosms at elevated CO2. Plant Pathol. 63 (5), 1033–1043. doi: 10.1111/ppa.12182

[B23] Kimura M. Tokai T. Takahashi-Ando N. Ohsato S. Fujimura M. (2007). Molecular and genetic studies of fusarium trichothecene biosynthesis: pathways, genes, and evolution. Biosci. Biotechnol. Biochem. 71 (9), 2105–2123. doi: 10.1271/bbb.70183 17827683

[B24] Klemsdal S. S. Elen O. (2006). Development of a highly sensitive nested-PCR method using a single closed tube for detection of fusarium culmorum in cereal samples. Lett. Appl. Microbiol. 42 (5), 544–548. doi: 10.1111/j.1472-765X.2006.01880.x 16620217

[B25] Lahti L. Shetty S. (2017). Microbiome r package.

[B26] Lysøe E. Harris L. J. Walkowiak S. Subramaniam R. Divon H. H. Riiser E. S. . (2014). The genome of the generalist plant pathogen fusarium avenaceum is enriched with genes involved in redox, signaling and secondary metabolism. PloS One 9 (11), e112703. doi: 10.1371/journal.pone.0112703 25409087 PMC4237347

[B27] Nicholson P. Simpson D. R. Weston G. Rezanoor H. N. Lees A. K. Parry D. W. . (1998). Detection and quantification ofFusarium culmorumandFusarium graminearumin cereals using PCR assays. Physiol. Mol. Plant Pathol. 53 (1), 17–37. doi: 10.1006/pmpp.1998.0170

[B28] Nicolaisen M. Supronienė S. Nielsen L. K. Lazzaro I. Spliid N. H. Justesen A. F. (2009). Real-time PCR for quantification of eleven individual fusarium species in cereals. J. Microbiol Methods 76 (3), 234–240. doi: 10.1016/j.mimet.2008.10.016 19047000

[B29] Nielsen L. K. Jensen J. D. Rodríguez A. Jørgensen L. N. Justesen A. F. (2012). TRI12 based quantitative real-time PCR assays reveal the distribution of trichothecene genotypes of f. graminearum and f. culmorum isolates in Danish small grain cereals. Int. J. Food Microbiol. 157 (3), 384–392. doi: 10.1016/j.ijfoodmicro.2012.06.010 22781579

[B30] Oghenekaro A. O. Oviedo-Ludena M. A. Serajazari M. Wang X. Henriquez M. A. Wenner N. G. . (2021). Population genetic structure and chemotype diversity of fusarium graminearum populations from wheat in Canada and north Eastern united states. Toxins 13 (3), 180. doi: 10.3390/toxins13030180 33804426 PMC7999200

[B31] Perry D. J. Lee S.-J. (2015). Identification of Canadian wheat varieties using OpenArray genotyping technology. J. Cereal Sci. 65, 267–276. doi: 10.1016/j.jcs.2015.08.002

[B32] R Core Team (2022). R: A language and environment for statistical computing.

[B33] Ripley B. (2011). “MASS: Support functions and datasets for Venables and ripley’s MASS,” in R package version, vol. 7. , 3–29.

[B34] Ruan Y. Babonich R. Clarke J. M. Hucl P. J. Clarke F. R. Knox R. E. . (2021). Differential reaction of hexaploid and tetraploid wheat to fusarium graminearum chemotypes in a controlled environment. Can. J. Plant Pathol. 43 (5), 760–768. doi: 10.1080/07060661.2021.1907447

[B35] Russell L. (2019). Emmeans: estimated marginal means, aka least-squares means. r package version 1.4. 3.01 (Iowa City, IA: The University of Iowa).

[B36] Shiferaw B. Smale M. Braun H.-J. Duveiller E. Reynolds M. Muricho G. (2013). Crops that feed the world 10. past successes and future challenges to the role played by wheat in global food security. Food Secur. 5 (3), 291–317. doi: 10.1007/s12571-013-0263-y

[B37] Simón M. R. Börner A. Struik P. C. (2021). Editorial: Fungal wheat diseases: Etiology, breeding, and integrated management. Front. Plant Sci. 12. doi: 10.3389/fpls.2021.671060 PMC8061728 33897751

[B38] Summerell B. A. Leslie J. F. (2006). “Species descriptions,” in The fusarium laboratory manual, (Ames, Iowa, USA: Blackwell Publishing Professional) 121–278.

[B39] Trail F. (2009). For blighted waves of grain: Fusarium graminearum in the postgenomics era. Plant Physiol. 149 (1), 103–110. doi: 10.1104/pp.108.129684 19126701 PMC2613717

[B40] Turkington T. K. Clear R. M. Demeke T. Lange R. Xi K. Kumar K. (2011). Isolation of fusarium graminearum from cereal, grass and corn residues from Alberta 2001–2003. Can. J. Plant Pathol. 33 (2), 179–186. doi: 10.1080/07060661.2011.560189

[B41] Turner A. S. Lees A. K. Rezanoor H. N. Nicholson P. (1998). Refinement of PCR-detection of fusarium avenaceum and evidence from DNA marker studies for phenetic relatedness to fusarium tricinctum. Plant Pathol. 47 (3), 278–288. doi: 10.1046/j.1365-3059.1998.00250.x

[B42] Valverde-Bogantes E. Bianchini A. Herr J. R. Rose D. J. Wegulo S. N. Hallen-Adams H. E. (2020). Recent population changes of fusarium head blight pathogens: drivers and implications. Can. J. Plant Pathol. 42 (3), 315–329. doi: 10.1080/07060661.2019.1680442

[B43] Varga E. Wiesenberger G. Hametner C. Ward T. J. Dong Y. Schöfbeck D. . (2015). New tricks of an old enemy: isolates of fusarium graminearum produce a type a trichothecene mycotoxin. Environ. Microbiol. 17 (8), 2588–2600. doi: 10.1111/1462-2920.12718 25403493 PMC4950012

[B44] Waalwijk C. van der Heide R. de Vries I. van der Lee T. Schoen C. Costrel-de Corainville G. . (2004). Quantitative detection of fusarium species in wheat using TaqMan. Eur. J. Plant Pathol. 110 (5), 481–494. doi: 10.1023/B:EJPP.0000032387.52385.13

[B45] Walkowiak S. Bonner C. T. Wang L. Blackwell B. Rowland O. Subramaniam R. (2015). Intraspecies interaction of fusarium graminearum contributes to reduced toxin production and virulence. Mol. Plant-Microbe Interact. 28 (11), 1256–1267. doi: 10.1094/MPMI-06-15-0120-R 26125491

[B46] Ward T. J. Clear R. M. Rooney A. P. O’Donnell K. Gaba D. Patrick S. . (2008). An adaptive evolutionary shift in fusarium head blight pathogen populations is driving the rapid spread of more toxigenic fusarium graminearum in north America. Fungal Genet. Biol. 45 (4), 473–484. doi: 10.1016/j.fgb.2007.10.003 18035565

[B47] Wickham H. (2016). “Data analysis,” in ggplot2 Vol. 189-201 (Springer).

[B48] Wickham H. (2017). Package ‘tidyr’. easily tidy data with’spread’and’gather ()’Functions.

[B49] Wickham H. (2019). Forcats: Tools for working with categorical variables (factors). version 0.4. 0.

[B50] Wickham H. Francois R. Henry L. Müller K. (2014). “dplyr,” in useR! conference.

[B51] Wilke C. O. (2019). Cowplot: streamlined plot theme and plot annotations for ‘ggplot2’ . R Package version 0.9 4.

[B52] Wilson A. Simpson D. Chandler E. Jennings P. Nicholson P. (2004). Development of PCR assays for the detection and differentiation of fusarium sporotrichioides and fusarium langsethiae. FEMS Microbiol. Lett. 233 (1), 69–76. doi: 10.1016/j.femsle.2004.01.040 15043871

[B53] Yli-Mattila T. Paavanen-Huhtala S. Parikka P. Konstantinova P. Gagkaeva T. (2004). Molecular and morphological diversity of fusarium species in Finland and north-Western Russia. Eur. J. Plant Pathol. 110, 573–585. doi: 10.1023/B:EJPP.0000032397.65710.69

