# Corrigendum: A survey of *Fusarium* species and ADON genotype on Canadian wheat grain

**DOI:** 10.3389/ffunb.2023.1271067

**Published:** 2023-08-30

**Authors:** Janice Bamforth, Tiffany Chin, Tehreem Ashfaq, Niradha Withana Gamage, Kerri Pleskach, Sheryl A. Tittlemier, Maria Antonia Henriquez, Shimosh Kurera, Sung-Jong Lee, Bhaktiben Patel, Tom Gräfenhan, Sean Walkowiak

**Affiliations:** ^1^ Canadian Grain Commission, Grain Research Laboratory, Winnipeg, MB, Canada; ^2^ Agriculture and Agri-Food Canada, Morden Research and Development Centre, Morden, MB, Canada; ^3^ University of Manitoba, Plant Science, Winnipeg, MB, Canada; ^4^ University of Manitoba, Microbiology, Winnipeg, MB, Canada; ^5^ Julius-Maximilian-University, Core Unit Systems Medicine, Würzburg, Bavaria, Germany

**Keywords:** *Fusarium*, wheat, deoxynivalenol (DON), chemotype, qPCR, grains

In the published article, there was an error in **Figure 5** as published. We received feedback that the number of crop districts listed in **Figure 5** for the province of Alberta was inconsistent with the number of districts described in Figure 1 (i.e. there are 7 districts but 8 are listed in **Figure 5**). Upon closer inspection, we identified an error where some samples were not correctly categorized according to their crop district. We have rectified the categorization of the samples into their correct crop districts. The change required us to generate a revised **Figure 5**. The corrected **Figure 5** and its caption appear below.

**Figure 5:**
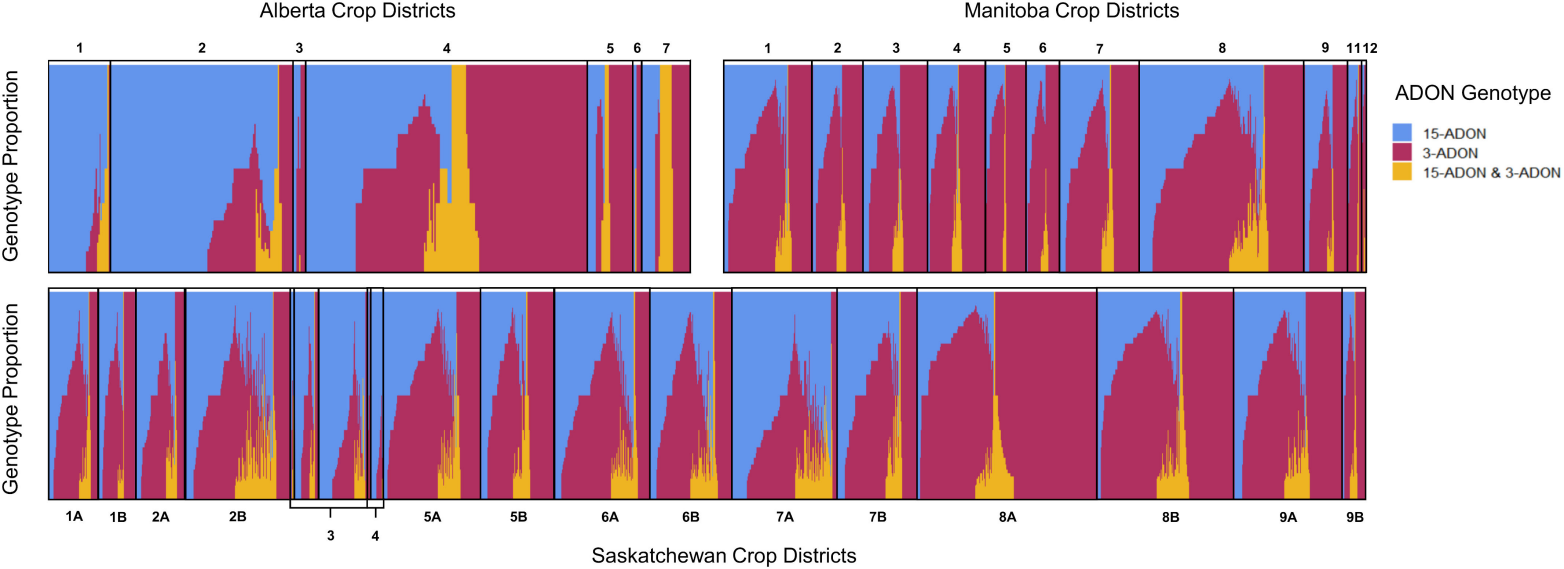
Proportion of ADON genotypes in each sample by western Canadian crop district. Each vertical bar represents a single sample where multiple FDK were tested. Samples corresponding to crop districts in Alberta (top left), Manitoba (top right), and Saskatchewan (bottom) contained kernels that were positive for 15-ADON (blue), 3-ADON (red), or both genotypes (yellow).

In the published article, there was an error in the **Supplementary Material**, *Supplementary Figures 1 and 2* and *Supplementary Table 2*. We identified an error where some samples were not correctly categorized according to their crop district. We have rectified the categorization of the samples into their correct crop districts. The change required *Supplementary Figures 1 and 2* and *Supplementary Table 2* to be revised in the original article.

The authors apologize for these errors and state that they do not change the scientific conclusions of the article in any way.

