# Supplementary material for: A survey of Fusarium species and ADON genotype on Canadian wheat grain
Source: Front Fungal Biol. 2022 Dec 2;3:1062444. doi: 10.3389/ffunb.2022.1062444 (PMC10512222; doi:10.3389/ffunb.2022.1062444)
Supplement: Supplementary file 1 [file DataSheet_1.docx]

**
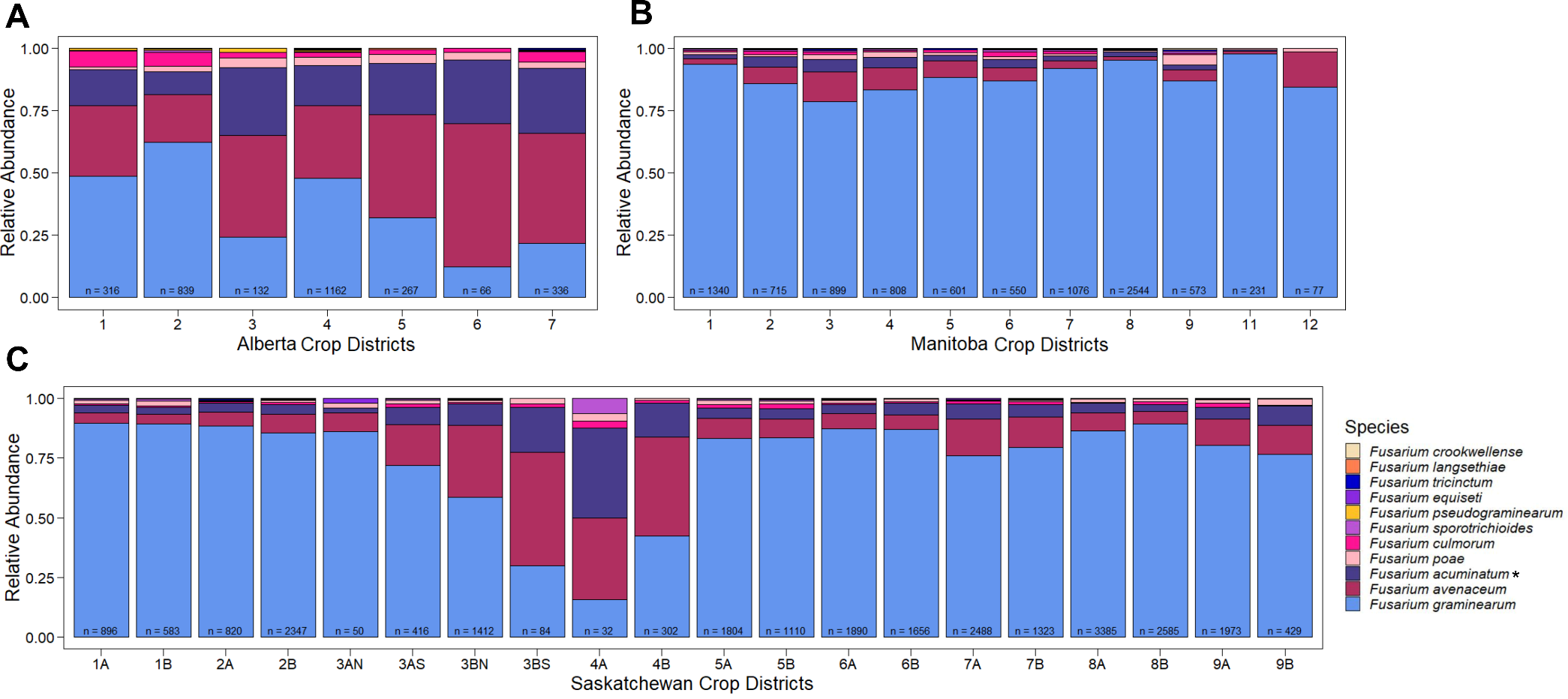
**

**Supplemental Figure 1.** Relative abundance of *Fusarium* species in kernels (n) across western Canadian crop districts across all years in study, in A) Alberta; B) Manitoba; C) Saskatchewan. Asterisks (*) signify putative *F. acuminatum*.

**
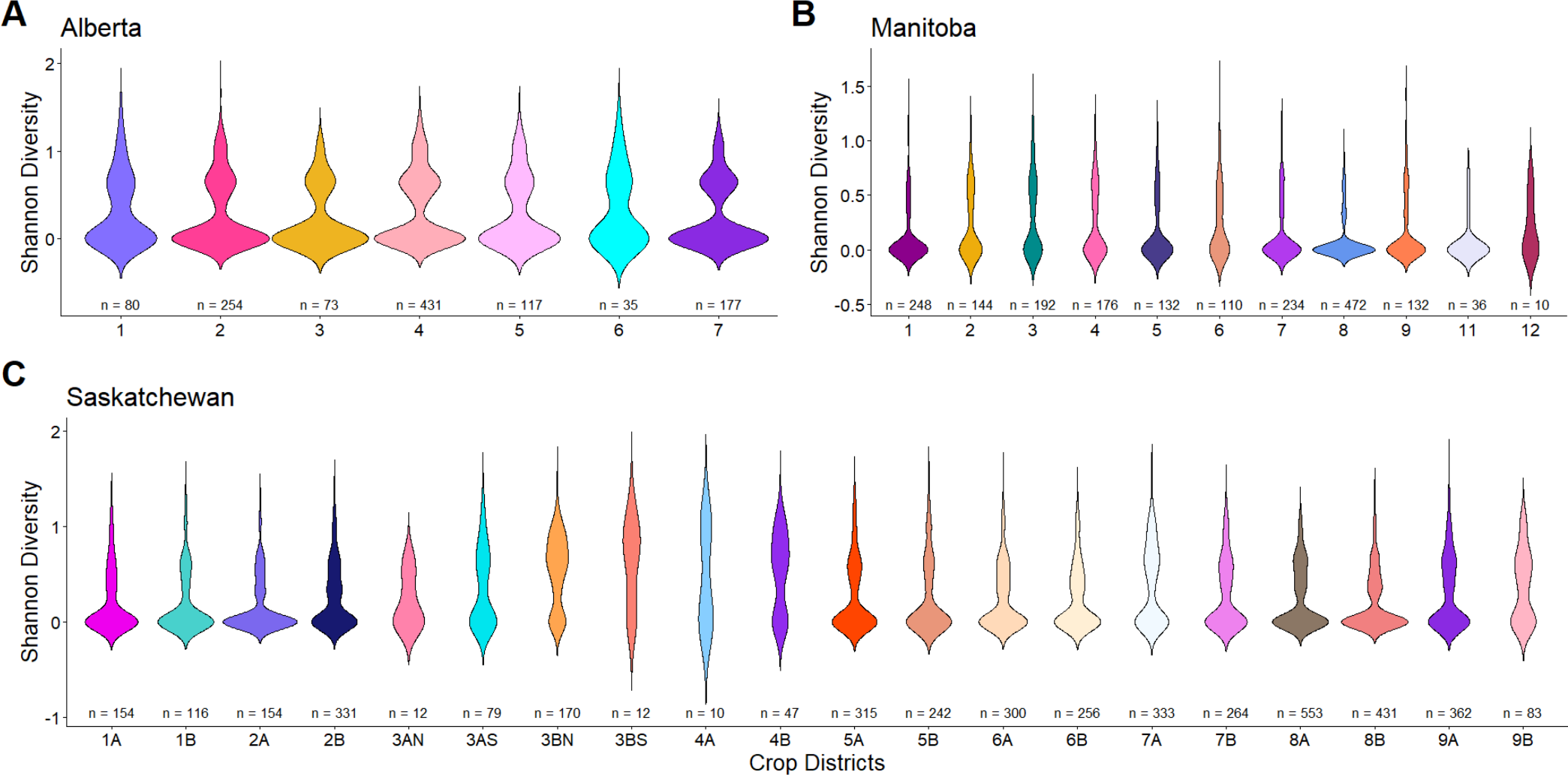
Supplemental Figure 2.** Species diversity of *Fusarium* species in wheat samples (n) across western Canadian crop districts across all years in study, in A) Alberta; B) Manitoba; C) Saskatchewan.


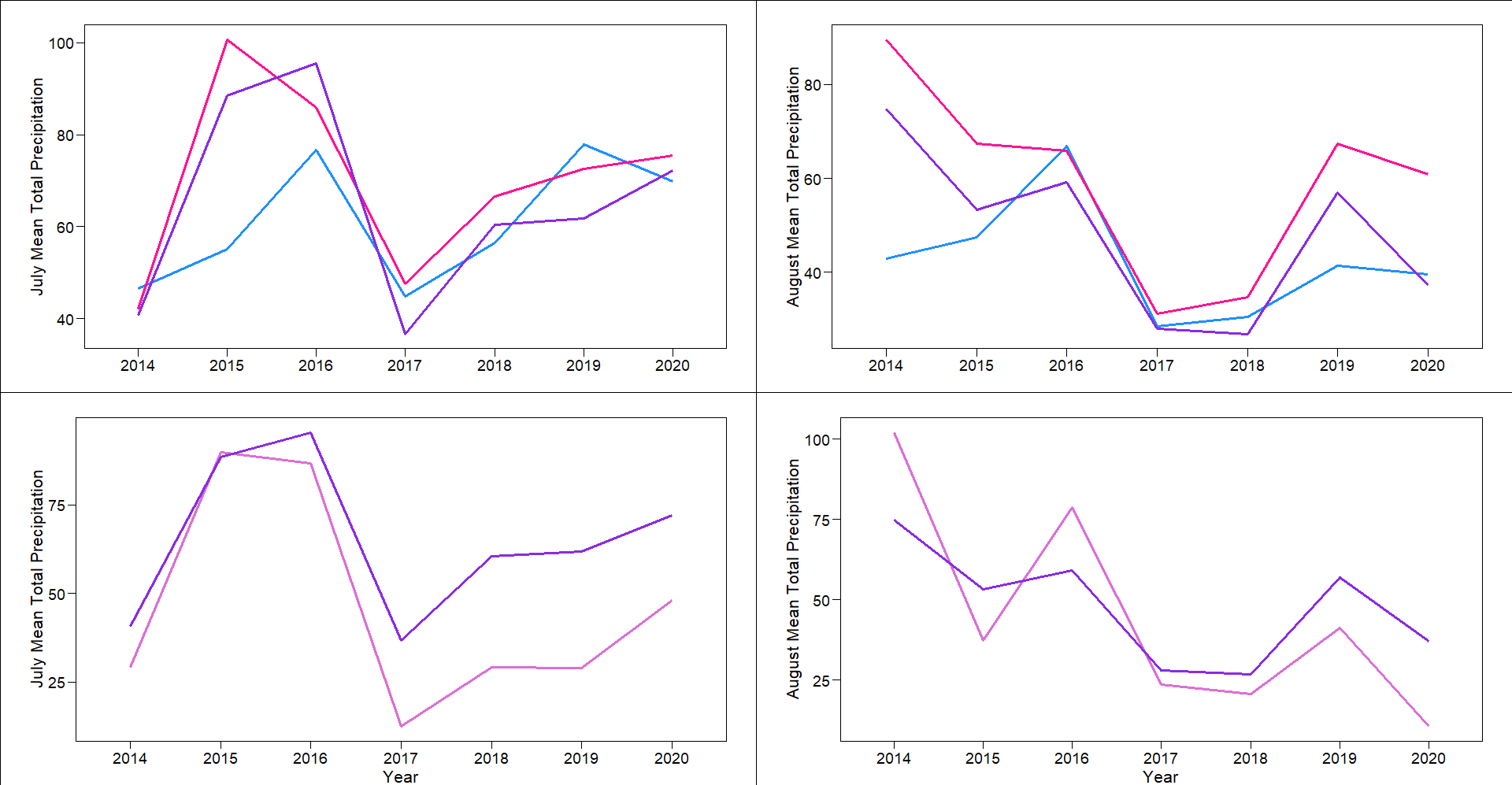

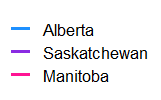

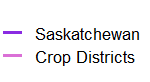


A)

B)

C)

D)

**Supplemental Figure 3.** Mean total precipitation from 2014-2020 (mm), in a) July for Alberta, Saskatchewan, and Manitoba, b) August for Alberta, Saskatchewan, and Manitoba, c) July for Saskatchewan and the southwestern crop districts of Saskatchewan, d) August for Saskatchewan and the southwestern crop districts of Saskatchewan.


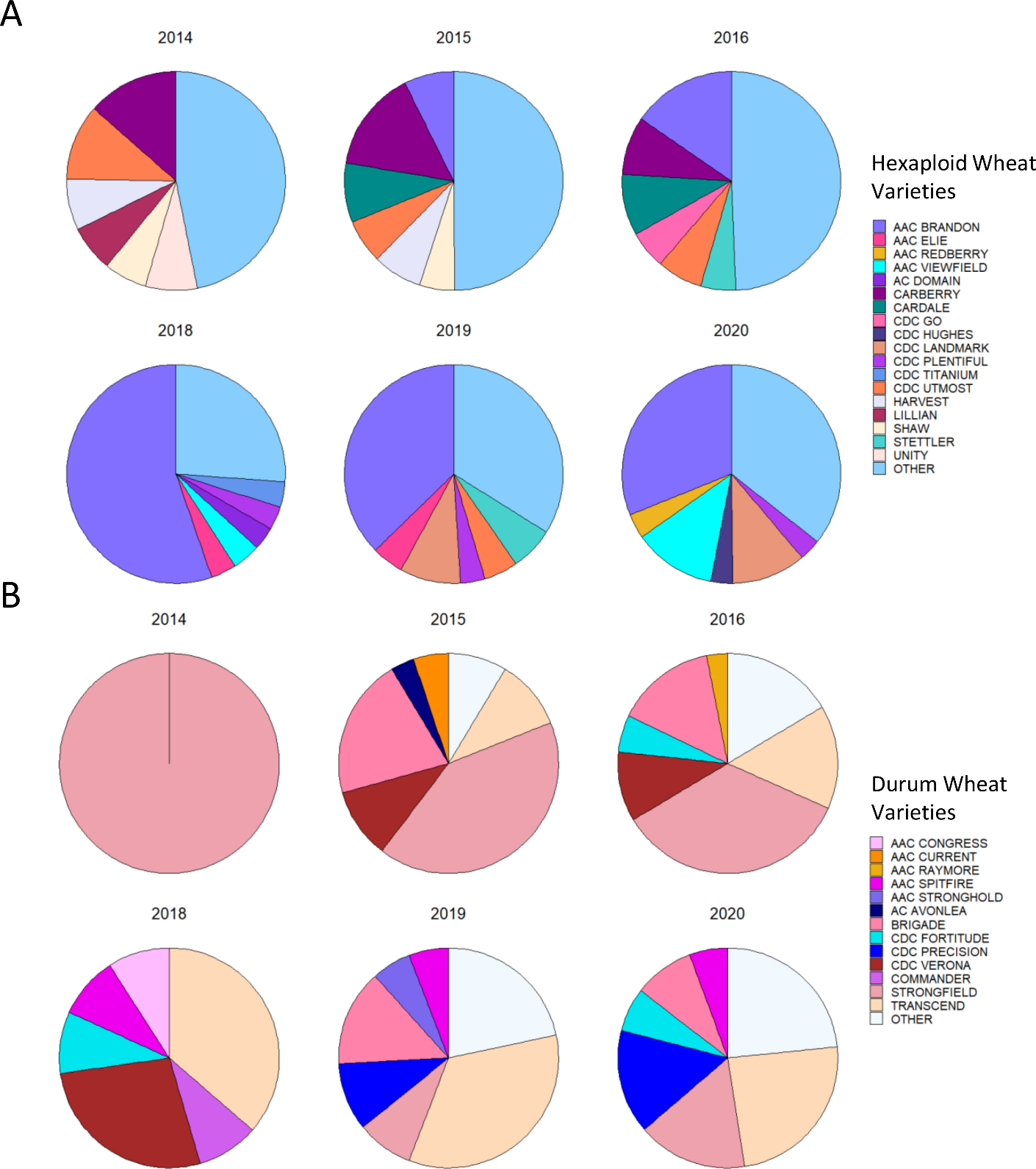


**Supplemental Figure 4.** Composition of varieties from 2014-2020 of samples in the study originating from a) hexaploid wheat and b) durum wheat.

**Table S1.** Correlation of *Fusarium* species presence, DON to %FHB severity. Asterisks (*) signify putative *F. acuminatum*.

|  | **By Kernel** | | **By Sample** | |
| --- | --- | --- | --- | --- |
| **Species** | **Kendall’s tau** | **p-value** | **Kendall’s tau** | **p-value** |
| *Fusarium acuminatum** | 0.024 | < 0.005 | 0.208 | < 0.005 |
| *Fusarium avenaceum* | 0.022 | < 0.005 | 0.203 | < 0.005 |
| *Fusarium crookwellense* | -0.010 | 0.006 | -0.005 | 0.567 |
| *Fusarium culmorum* | -0.033 | < 0.005 | -0.001 | 0.916 |
| *Fusarium equiseti* | 0.003 | 0.396 | 0.038 | < 0.005 |
| *Fusarium graminearum* | 0.145 | < 0.005 | 0.509 | < 0.005 |
| *Fusarium langsethiae* | -0.003 | 0.397 | 0.001 | 0.935 |
| *Fusarium poae* | -0.026 | < 0.005 | 0.031 | 0.001 |
| *Fusarium pseudograminearum* | -0.007 | 0.045 | 0.013 | 0.182 |
| *Fusarium tricinctum* | -0.007 | 0.057 | 0.019 | 0.044 |
| *Fusarium sporotrichioides* | -0.000 | 0.888 | 0.05 | < 0.005 |
| DON | 0.272 | < 0.005 | 0.488 | < 0.005 |

**Table S2.** Differences in species presence by year, province, year by province, and by western crop districts by generalized linear regression fitted with a Poisson distribution and log link function, and a one-way ANOVA. Asterisks (*) signify putative *F. acuminatum*.

| **Species** | **Year** | **Province** | **Year * Province** | **Western Crop Districts** |
| --- | --- | --- | --- | --- |
| *Fusarium acuminatum** | p < 0.0001 | p < 0.0001 | p < 0.0001 | p < 0.0001 |
| *Fusarium avenaceum* | p < 0.0001 | p < 0.0001 | p < 0.0001 | p < 0.0001 |
| *Fusarium crookwellense* | p < 0.0001 | p < 0.0001 | p = 0.9935 | p = 0.9164 |
| *Fusarium culmorum* | p < 0.0001 | p < 0.0001 | p < 0.0001 | p < 0.0001 |
| *Fusarium equiseti* | p < 0.0001 | p < 0.0001 | p < 0.0001 | p = 0.0292 |
| *Fusarium graminearum* | p < 0.0001 | p < 0.0001 | p < 0.0001 | p < 0.0001 |
| *Fusarium langsethiae* | p = 0.2085 | p = 0.2544 | p = 1 | p = 1 |
| *Fusarium poae* | p < 0.0001 | p < 0.0001 | p < 0.0001 | p < 0.0001 |
| *Fusarium pseudograminearum* | p = 0.002 | p < 0.0001 | p = 0.544 | p = 0.0043 |
| *Fusarium sporotrichioides* | p < 0.0001 | p < 0.0001 | p < 0.0001 | p < 0.0001 |
| *Fusarium tricinctum* | p < 0.0001 | p < 0.0001 | p = 0.025 | p = 0.0742 |

**Table S3.** Mean Total Precipitation in Alberta, Saskatchewan, Manitoba, and southwestern Saskatchewan crop districts from July and August 2014-2020.

| Year | Location | July Mean Total Precipitation | August Mean Total Precipitation |
| --- | --- | --- | --- |
| 2014 | Alberta | 46.55 | 42.85 |
|  | Saskatchewan | 40.71 | 74.72 |
|  | Manitoba | 42.14 | 89.53 |
|  | Saskatchewan Crop Districts | 29.09 | 102.05 |
| 2015 | Alberta | 55.14 | 47.36 |
|  | Saskatchewan | 88.42 | 53.32 |
|  | Manitoba | 100.63 | 67.42 |
|  | Saskatchewan Crop Districts | 89.94 | 37.46 |
| 2016 | Alberta | 76.67 | 66.97 |
|  | Saskatchewan | 95.47 | 59.17 |
|  | Manitoba | 86.01 | 65.91 |
|  | Saskatchewan Crop Districts | 86.73 | 78.64 |
| 2017 | Alberta | 44.89 | 28.48 |
|  | Saskatchewan | 36.70 | 28.06 |
|  | Manitoba | 47.62 | 31.13 |
|  | Saskatchewan Crop Districts | 12.43 | 23.68 |
| 2018 | Alberta | 56.40 | 30.43 |
|  | Saskatchewan | 60.41 | 26.88 |
|  | Manitoba | 66.56 | 34.70 |
|  | Saskatchewan Crop Districts | 29.02 | 20.64 |
| 2019 | Alberta | 77.82 | 41.33 |
|  | Saskatchewan | 61.83 | 57.04 |
|  | Manitoba | 72.54 | 67.39 |
|  | Saskatchewan Crop Districts | 28.97 | 41.17 |
| 2020 | Alberta | 69.88 | 39.52 |
|  | Saskatchewan | 72.15 | 37.20 |
|  | Manitoba | 75.41 | 60.89 |
|  | Saskatchewan Crop Districts | 47.97 | 10.65 |

**Table S4**. Varieties of hexaploid and durum wheat that were sampled (n) in this study from 2014-2020.

| Wheat Type | Year | Variety | n |
| --- | --- | --- | --- |
| Hexaploid Wheat | 2014 | CARBERRY | 194 |
|  |  | CDC UTMOST | 161 |
|  |  | UNITY | 110 |
|  |  | HARVEST | 109 |
|  |  | LILLIAN | 98 |
|  |  | SHAW | 91 |
|  |  | OTHER | 673 |
|  | 2015 | CARBERRY | 233 |
|  |  | CARDALE | 136 |
|  |  | AAC BRANDON | 114 |
|  |  | HARVEST | 113 |
|  |  | CDC UTMOST | 101 |
|  |  | SHAW | 81 |
|  |  | OTHER | 774 |
|  | 2016 | AAC BRANDON | 107 |
|  |  | CARDALE | 63 |
|  |  | CARBERRY | 60 |
|  |  | CDC UTMOST | 47 |
|  |  | CDC GO | 39 |
|  |  | STETTLER | 36 |
|  |  | OTHER | 343 |
|  | 2018 | AAC BRANDON | 146 |
|  |  | AAC VIEWFIELD | 11 |
|  |  | CDC TITANIUM | 10 |
|  |  | AAC ELIE | 10 |
|  |  | CDC PLENTIFUL | 9 |
|  |  | AC DOMAIN | 9 |
|  |  | OTHER | 69 |
|  | 2019 | AAC BRANDON | 452 |
|  |  | CDC LANDMARK | 109 |
|  |  | STETTLER | 78 |
|  |  | CDC UTMOST | 61 |
|  |  | AAC ELIE | 57 |
|  |  | CDC PLENTIFUL | 44 |
|  |  | OTHER | 411 |
|  | 2020 | AAC BRANDON | 298 |
|  |  | AAC VIEWFIELD | 117 |
|  |  | CDC LANDMARK | 105 |
|  |  | AAC REDBERRY | 35 |
|  |  | CDC PLENTIFUL | 31 |
|  |  | CDC HUGHES | 31 |
|  |  | OTHER | 341 |
| Durum Wheat | 2014 | STRONGFIELD | 1 |
|  | 2015 | STRONGFIELD | 24 |
|  |  | BRIGADE | 12 |
|  |  | CDC VERONA | 6 |
|  |  | TRANSCEND | 6 |
|  |  | AAC CURRENT | 3 |
|  |  | AC AVONLEA | 2 |
|  |  | OTHER | 5 |
|  | 2016 | STRONGFIELD | 146 |
|  |  | TRANSCEND | 64 |
|  |  | BRIGADE | 62 |
|  |  | CDC VERONA | 43 |
|  |  | CDC FORTITUDE | 23 |
|  |  | AAC RAYMORE | 13 |
|  |  | OTHER | 69 |
|  | 2018 | TRANSCEND | 4 |
|  |  | CDC VERONA | 3 |
|  |  | AAC CONGRESS | 1 |
|  |  | AAC SPITFIRE | 1 |
|  |  | CDC FORTITUDE | 1 |
|  |  | COMMANDER | 1 |
|  | 2019 | TRANSCEND | 41 |
|  |  | BRIGADE | 14 |
|  |  | CDC PRECISION | 12 |
|  |  | STRONGFIELD | 10 |
|  |  | AAC SPITFIRE | 7 |
|  |  | AAC STRONGHOLD | 7 |
|  |  | OTHER | 26 |
|  | 2020 | TRANSCEND | 30 |
|  |  | STRONGFIELD | 20 |
|  |  | CDC PRECISION | 19 |
|  |  | BRIGADE | 11 |
|  |  | CDC FORTITUDE | 8 |
|  |  | AAC SPITFIRE | 7 |
|  |  | OTHER | 29 |
